# The effect of accelerated pulsed high-fluence corneal cross-linking on corneal endothelium; a prospective specular microscopy study

**DOI:** 10.1186/s12886-023-02912-6

**Published:** 2023-04-18

**Authors:** Mahmoud Abdel-Radi, Naglaa Abdelmohsen, Hazem Abdelmotaal, Mohamed Tarek Abd El-Moneim

**Affiliations:** Department of Ophthalmology, Assiut University, Assiut University Hospital 6th floor, Assiut, 71516 Egypt

**Keywords:** Cross-linking, CXL, Accelerated CXL, Pulsed light, pl-ACXL, Specular microscopy

## Abstract

**Background:**

Corneal collagen cross-linking (CXL) is a procedure utilized for halting keratoconus progression with different approved protocols. The current study aimed to assess the corneal endothelial changes following the relatively new accelerated pulsed high-fluence protocol of epithelium-off corneal cross-linking for the treatment of mild to moderate keratoconus.

**Methods:**

This prospective case series study enrolled 45 eyes of 27 patients with mild to moderate progressive keratoconus who underwent accelerated pulsed high-fluence CXL (pl-ACXL, 30 mW/ cm^2^ UVA at 365 nm wavelength, 8 min pulsed mode 1 s on / 1 s off with a total energy of 7.2 J/ cm^2^). The main outcome measures were corneal endothelial changes assessed by specular microscopy at 3 and 6 months postoperatively including endothelial cell density (ECD), coefficient of variation, percentage of hexagonal cells, average, minimum and maximum endothelial cell sizes. Demarcation line depth was assessed 1 month following surgery.

**Results:**

The mean age of the studied sample was 24.89 ± 7.21. The mean preoperative ECD (2944.6 ± 247.41 cell/mm^2^) showed non-significant reduction at 3 and 6 months postoperatively (2931.03 ± 253.82 and 2924.7 ± 224.88 cell/mm^2^, respectively, P-value = 0.361). There were no significant changes in the mean coefficient of variation, percentage of hexagonal cells, average, minimum and maximum endothelial cell sizes at 3 and 6 months following pl-ACXL (P-value > 0.05). The mean demarcation line depth 1 month after pl-ACXL was 214 ± 17.43 μm.

**Conclusions:**

Corneal endothelial changes following accelerated pulsed high-fluence CXL were minimal with stability of endothelial cell count and non-significant morphological changes.

**Trial Registration:**

Clinicaltrials.gov: NCT04160338 (13/11/2019).

## Background

Corneal collagen cross-linking (CXL) is a well-established procedure for halting progression of keratoconus [1, 2]. CXL increases the stiffness and enhances the rigidity of the ectatic cornea through the chemical interaction of ultraviolet-A radiation (315–400 nm) and riboflavin to induce covalent bond formation between the corneal collagen fibers [3–5]. The standard CXL protocol (known also as the Dresden protocol) was initially introduced with an ultraviolet-A (UVA) intensity of 3 mW/cm^2^ at 370 nm and an exposure time of 30 min [6]. Accelerated CXL (ACXL) protocols were based on the Bunsen-Roscoe law of reciprocity through decreasing UVA exposure time by increasing UVA fluency to obtain the same overall total UVA dosage with two available modes, the pulsed light (pl-ACXL)and the continuous light (cl-ACXL) accelerated modes [7, 8].

One of the main concerns about the safety of accelerated epithelium-off CXL protocols is the possible endothelial damage with subsequent corneal decompensation due to higher levels of UVA irradiance compared with the standard protocol [9]. In a preliminary report, Gatzioufas et al. found that high-fluence CXL at 18mW/cm^2^ didn’t inversely affect the corneal endothelial cell count or epithelial regeneration [10]. Thereafter, Seiler et al. [11] and Mazzotta et al. [12] documented the safety of accelerated higher fluence pl-ACXL on corneal endothelium. Oxygen plays a vital role in the success of collagen crosslinking reaction. Continuous UVA illumination causes rapid depletion of oxygen in a riboflavin-soaked cornea and hence the idea of pl-ACXL was invented based on the fact that alternate turning of the UV light on and off leads to replenishment of the oxygen back to its original level [13]. Pulsed light ACXL treatment optimizes intraoperative oxygen availability thus increasing the efficiency of high fluence accelerated CXL compared with continuous light treatment [14]. Ultraviolet irradiation used in CXL stimulates the formation of cytotoxic free radicals and apoptosis in keratocytes and corneal endothelial cells [15]. It is believed that soaking the cornea with riboflavin before UVA exposure can markedly reduce these cytotoxic effects thus protecting the corneal endothelium and deeper ocular structures [16].

Previous studies assessed the safety of the standard conventional or continuous light accelerated CXL protocols on the corneal endothelium [17, 18]. Few studies assessed the corneal endothelial changes following pulsed high-fluence accelerated CXL and they focused mainly on changes in the endothelial cell count [19, 20]. Our study assessed the detailed morphological data of corneal endothelium after pulsed-light high fluence accelerated crosslinking using 30mW/cm^2^ power (1 s on/1 sec off) for 8 min of UV-A exposure time.

## Patients and methods

### Study design and settings

This is a prospective uncontrolled case series study involving patients with KC who underwent epithelium-off high-fluence pulsed light accelerated CXL. The study was carried out at El-Nour eye center (private practice), Assiut, Egypt.

### Ethical approval

The study was approved by the Institutional Review Ethics Committee of the Faculty of Medicine at Assiut University (IRB: 17,101,014) and was conducted under the Declaration of Helsinki. Clinical trials registry at clinicatrials.gov: NCT04160338 (13/11/2019). Every patient was informed about his or her condition, the nature of the CXL procedure, its possible complications and written informed consent was obtained was obtained from each patient or the parents if the patient was below 18 years old.

### Patient selection

Patients included in the study were those with mild to moderate progressive keratoconus of Kmax less than 56 diopters and corneal pachymetry (thinnest location) more than 400 μm. Progression of keratoconus was documented by increase of one diopter (D) or more in Kmax or steepest keratometry or manifest cylinder over 6-months and/or deterioration of CDVA of 2 lines or more over 12 months. Exclusion criteria were patients with thin corneas of less than 400 μm, corneal scars, spring catarrh, previous corneal surgeries such as intrastromal corneal ring segments and systemic autoimmune diseases.

### Preoperative assessment & keratoconus diagnosis

A full ophthalmological examination was done including assessment of uncorrected and corrected distance visual acuities (UCVA, CDVA), auto-keratorefraction, slit-lamp biomicroscopy, intraocular pressure and examination of anterior and posterior segments. Diagnosis of keratoconus and its progression was determined by using Scheimpflug imaging (Pentacam, WaveLight Oculyzer II, Germany) under scotopic conditions with proper quality to avoid examiner-dependent variability measuring astigmatism, corneal topography, K-max, pachymetric map, corneal posterior & anterior elevation maps, Belin / Ambrósio enhanced ectasia display. One ophthalmologist performed the preoperative assessment and pentacam evaluation.

### Specular microscopy

CEM-530 (Nidek Co, Ltd, Gamagori, Japan) was the non-contact specular microscope used to evaluate the endothelial cell density (ECD), the coefficient of variation in cell size (CV), the percentage of hexagonal cells (HEX), minimum endothelial cell size (Min), maximum endothelial cell size (Max) and average endothelial cell size (AVG). Sixteen endothelial photographs of a corneal endothelial surface area can be captured per scan using the auto-alignment function with a central fixation target. The best-quality micrographs were analyzed. A fully automated system measures a corneal endothelial area of 0.25 × 0.55 mm and then automatically calculates the previously mentioned endothelial cell parameters by built-in software. Specular microscopy evaluation was conducted by one operator.

### The surgical procedure of accelerated pulsed high-fluence corneal cross-linking

Topical pilocarpine hydrochloride 2% and moxifloxacin 0.5% were instilled 1 h before starting the procedure. Preservative-free topical anesthetic was used followed by painting the eyelids and eyelashes with 10% povidone-iodine solution and sterile draping. Our patient was placed in a supine position comfortably with insertion of the lid speculum. The central 8 mm corneal epithelium was removed by mechanical debridement using a blunt Hockey knife. The room lights were turned off to protect riboflavin. Riboflavin 0.1% (vitamin B2) in hydroxypropyl methylcellulose (VibeX Rapid®, Avedro Inc., USA) was applied to cover the cornea completely every 2 min for 10 min. The patient’s cornea was exposed to pulsed light accelerated CXL using the KXL system (Avedro, Inc., Waltham, MA, USA). The procedure settings were 30 mW/ cm^2^ UVA at 365 nm wavelength was applied to the cornea for 8 min pulsed mode (1 s on / 1 s off) with a total energy dose of 7.2 J/ cm^2^. Balanced salt solution (BSS, Alcon Lab., Fort Worth, TX, USA) was used to wet the cornea as needed during ultraviolet irradiation. At the end of the procedure, the cornea was rinsed with chilled BSS and a soft bandage contact lens was applied. Protection of the surgeon’s eyes was done using protective goggles. Protection of the limbal stem cells during UVA treatment was achieved by accurate focusing and meticulous centration of the UV circle. One ophthalmic surgeon performed all CXL surgeries.

### Postoperative care

Our postoperative treatment protocol for pl-ACXL was the prescription of topical moxifloxacin 0.5% five-times daily for 2 weeks, Fluorometholone 0.1% twice daily for a month, preservative-free lubricants for 3 months and oral NSAID analgesics for the first few postoperative days. Soft bandage contact lens was removed after complete epithelial healing.

### Follow-up and outcome measures

Specular microscopy was performed to evaluate the endothelial changes following pl-ACXL at 3 and 6 months postoperatively. Anterior segment ocular coherence tomography (AS-OCT, Spectralis SD-OCT (Heidelberg GmbH, Germany) was performed 1 month after pl-ACXL to measure the demarcation line depth. The postoperative assessment was done by the same ophthalmologist who performed the preoperative assessment using the same tools under the same settings.

### Statistical analysis

Statistical analysis was performed with IBM® SPSS® Statistics version 26 for Windows. The Shapiro-Wilk test was used to determine compliance of the data to normal distribution. Categorical variables were described by number and percentage where continuous variables were expressed as mean ± standard deviation (SD) for parametric variables or mean ± standard error (SE) for nonparametric variables. Differences between more than two periods were measured using the repeated measures ANOVA test while post hoc test was used for pairwise comparison with Bonferroni correction. Pearson correlation coefficient was used to explore the correlation between preoperative pachymetry or preoperative Kmax and postoperative endothelial cell loss. The level of significance was set at P value < 0.05.

## Results

### Demographic and baseline preoperative data

The study included 45 eyes of 27 patients with a mean age of 24.89 ± 7.21 years. The demographic and preoperative baseline characteristics are illustrated in Table 1. The mean baseline endothelial cell density of the studied sample as measured by specular microscopy was 2944.60 ± 247.405 cell/mm^2^.

**Table 1:** Demographic and baseline preoperative data

Parameter	Value
No. of Eyes	45
No. of Patients	27
Age (years) † (mean ± SE)	24.89 ± 7.21 (16–40)
Gender (Female/Male)	17 / 10
logMAR UDVA †(mean ± SE)	0.74 ± 0.08 (0.2 − 0.1.5)
logMAR CDVA † (mean ± SD)	0.31 ± 0.05 (0–1)
MRSE (D) † (mean ± SE)	-4.25 ± 0.80 (+ 1.25 to -11.0)
Pachymetry µm (Pachy Apex) † (mean ± SD)	478.59 ± 45.30 (413–594)
Pachymetry µm (Thinnest location) † (mean ± SD)	467.67 ± 36.97 (401–553)
Keratometry (D) (Kmax) † (mean ± SE)	50.80 ± 3.43 (45.1–56)
Endothelial cell density (cell/mm^2^)† (mean ± SD)	2944.60 ± 247.40 (2454–3468)

† Data expressed as Mean ± SD/ SE, Range. CDVA = Corrected distance visual acuity, D = Diopters, Kmax = maximum keratometry, logMAR = logarithm of the minimum angle of resolution, MRSE = manifest refraction spherical equivalent, UDVA = Uncorrected distance visual acuity.

### Endothelial cell changes by specular microscopy

The endothelial changes observed after pl-ACXL at 3 and 6 months postoperatively are summarized in Table 2.

**Fig. 1:**
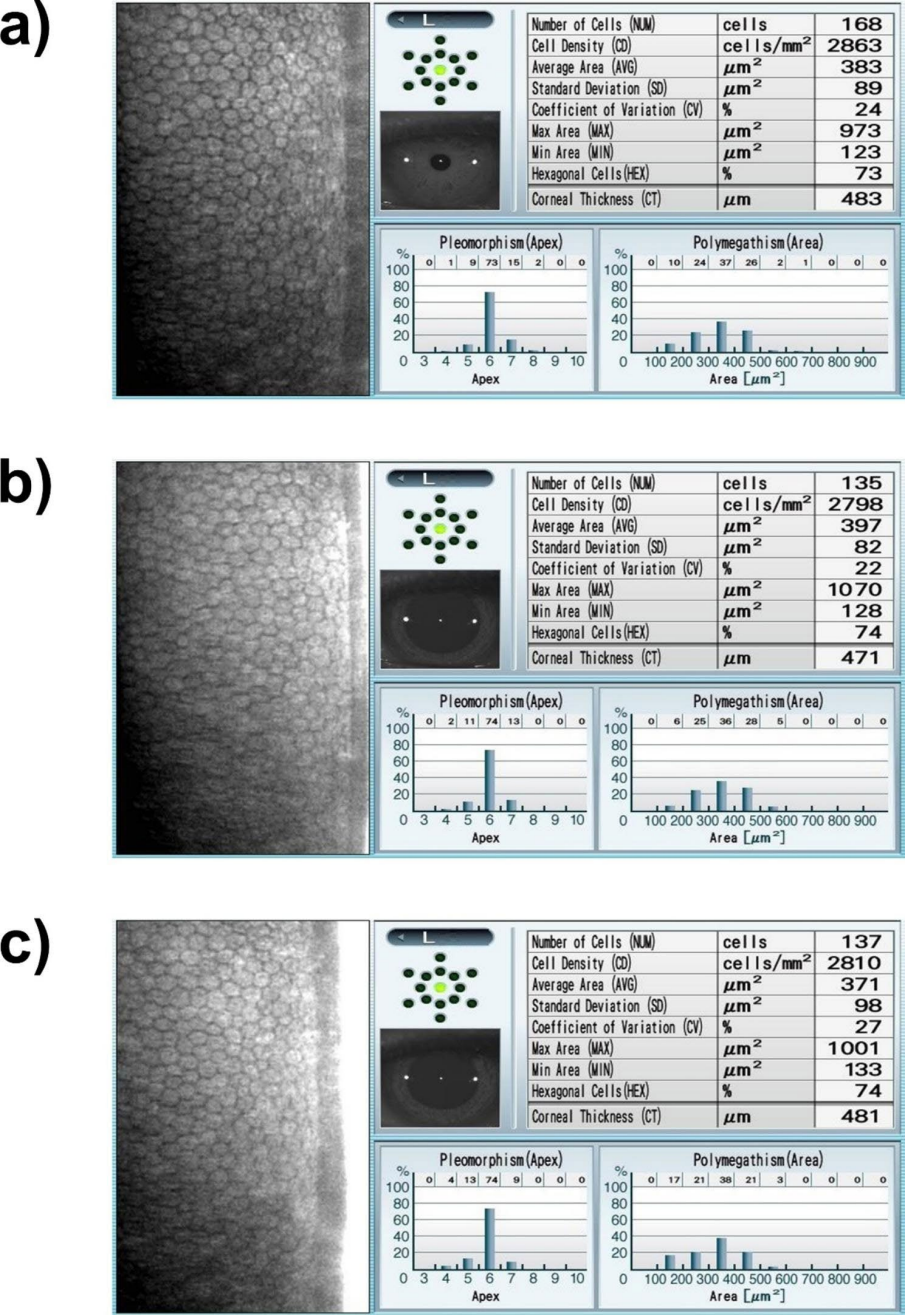
Corneal endothelial changes following accelerated pulsed high-fluence cross-linking. Legend: Specular microscopy images of a 28-year-old male patient with keratoconus who underwent accelerated pulsed high-fluence cross-linking. (a) Preoperative, (b) 3 months postoperatively, (c) 6 months postoperatively. AVG, average; CD, cell density; CT, corneal thickness; CV, coefficient of variance; Max, maximum; Min, minimum; SD, standard deviation

The mean preoperative ECD was 2944.6 ± 247.41 cell/mm^2^. A non-significant reduction of the mean ECD was observed at 3 and 6 months (2931.03 ± 253.82 and 2924.7 ± 224.88, respectively). The overall change of the mean ECD at the end of follow-up period was statistically non-significant (P-value = 0.361). Figure 1 illustrates specular microscopy images of one of the enrolled keratoconic patients before and after treatment with accelerated pulsed high-fluence CXL.

The mean coefficient of variation (CV) % showed non-significant change throughout the study (P-value = 0.34). The mean percentage of hexagonal cells (pleomorphism) showed a non-significant change throughout the study (P-value = 0.053). However, there was a significant percentage increase at 6 months compared with the 3rd-month results (P-value = 0.01).

No significant changes were observed regarding the mean average endothelial cell size (P = 0.304), the mean minimum endothelial cell size (P = 0.230) and the mean maximum endothelial cell size (P = 0.063).

**Table 2:** Specular microscopy parameters before and at 3 and 6 months after cross-linking

Variables	Preoperative	3 months Postop.	6 months Postop.	P-value*	P ^a^	P ^b^	P ^c^
**Endothelial cell density (cell/mm** ^**2**^ **)†**	2944.6 ± 247.41	2931.03 ± 253.82	2924.7 ± 224.88	0.361	0.760	0.410	0.983
**Coefficient of variation (CV)% †**	28.27 ± 5.32	29.07 ± 4.86	28.07 ± 3.97	0.340	0.242	0.811	0.124
**Hexagonal cells percentage%†**	66.3 ± 5.38	64.97 ± 7.02	67.1 ± 6.49	0.053	0.262	0.277	0.01**
**Average Endothelial cell size µm** ^**2**^ **†**	342.07 ± 29.54	338.9 ± 27.53	343.7 ± 27.27	0.304	0.337	0.589	0.138
**Minimum Endothelial cell size µm** ^**2**^ **†**	108.03 ± 6.98	113.3 ± 3.74	116.4 ± 4.48	0.230	0.422	0.132	0.558
**Maximum Endothelial cell size µm** ^**2**^ **†**	999.1 ± 236.15	1054.37 ± 243.27	1019.5 ± 243.67	0.063	0.054	0.062	0.12

**†**Data expressed as Mean ± SD (Standard deviation) * Repeated measures ANOVA test Post hoc test was used for pairwise comparison with Bonferroni correction: P ^a^: Preoperative vs. 3 months postop.

P ^b^: Preoperative vs. 6 months postop.

P ^c^: Postoperative 3 months vs. 6 months.

** Statistical significance.

### Demarcation line

**Fig. 2:**
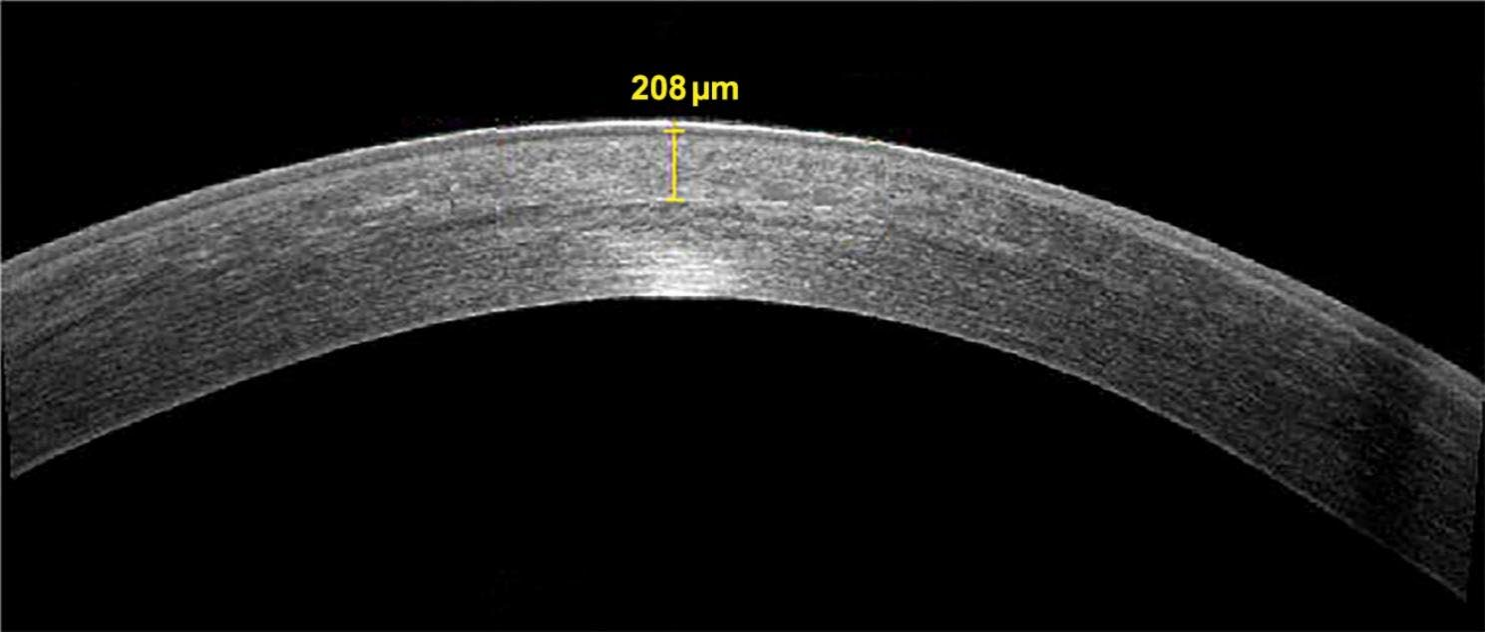
Demarcation line depth following accelerated pulsed high-fluence cross-linking. Legend: Anterior segment ocular coherence tomography corneal scan of a 21-year old female patient with keratoconus showing demarcation line depth 1-month following accelerated pulsed high-fluence cross-linking

The mean demarcation line depth 1 month after pl-ACXL was 214 ± 17.43 μm **(**Fig. 2**)** with a mean percentage of demarcation line depth of 44.34 ± 9.54% (Percentage of the volume of the cross-linked cornea in relation to the preoperative central corneal thickness).

### Correlations

A correlation between the preoperative pachymetry at the thinnest location and the postoperative endothelial cell loss at 6 months was explored. A negative correlation was observed but it was of no statistical significance (Pearson correlation coefficient r = -0.049, P-value = 0.796). Another correlation between the preoperative Kmax and the postoperative endothelial cell loss at 6 months was explored and a positive correlation was observed but it was also of no statistical significance (P-value of 0.684).

## Discussion

The safety profile of corneal collagen cross-linking is of great concern for both ophthalmologists and keratoconus patients. The current study is one of few studies that reported detailed corneal endothelial changes by specular microscopy after pulsed high-fluence accelerated epithelium-off CXL.

Seiler et al. [11] studied the effect of customized irradiation by using 9mW/cm^2^ with varying total energy ranging from 5.4 J/cm² up to 10 J/cm^2^ in 20 eyes of 20 patients with progressive keratoconus and concluded that no significant changes were observed in the endothelial cell count. Mazzotta et al. [12] studied the morphological and clinical effects of high fluence pulsed light topography-guided UV-A energy emission by using 30mW/cm^2^ of pulsed irradiation with varying total energy levels ranging from 7.2 J/cm^2^ up to 15 J/cm^2^ on the cornea and demonstrated that no endothelial damage was observed in terms of morphology and cell count.

The current study showed a slight non-significant reduction of ECD at both 3 and 6 months after pl-ACXL which reflects overall stability in ECD. Other specular microscopy morphological parameters including the mean CV, hexagonal cell percentage, the average endothelial cell size, the minimum and the maximum endothelial cell size showed non-significant changes at 3 and 6 months postoperatively. It.

Our specular microscopy results were consistent with Gore et al. [19] who evaluated ECD at baseline, 6, 12, and 24 months following pl-ACXL using the same KXL System (Avedro, Inc., Waltham, MS, USA) and protocol utilized in our study and reported stable ECD at baseline, 6, 12, and 24 months postoperatively in patients with Kmax < 55 D. Some studies [13, 20] compared ECD following the standard conventional CXL or cl-ACXL versus pl-ACXL and reported no postoperative endothelial damage in these different CXL protocols.

In a fellow-eye study design, Cingü et al. [17] utilized an accelerated continuous-light CXL protocol of instilling iso-osmolar 0.1% riboflavin solution without dextran every 3 min for 30 min followed by 5 min of 18 mW/cm^2^ UVA irradiation and concluded that the mean ECD values in treated eyes were significantly decreased in 1st week, at 1st month, and in 3rd month of follow-up and the mean percentages of the hexagonality significantly decreased and CV significantly increased in 1st week and at 1st month. However, all the endothelial parameters in their study recovered at 6 months in treated eyes when compared with their preoperative values or between the measurements of treated and fellow eyes. They suggested that the early transient endothelial changes observed may be due to higher energy delivery into the deeper stromal layers caused by increased riboflavin saturation as well as the more intense UVA irradiance. Badawi [18] studied 40 eyes with progressive keratoconus and 10 eyes with post-LASIK ectasia treated with continuous light ACXL (10 mW/cm^2^ for 9 min). Corneal endothelial parameters were assessed 3, 6, and 12 months after CXL using specular microscopy (Tomy EM-3000). The study concluded that accelerated CXL might have a transient negative impact on endothelial cell density and/or endothelial morphology especially at 3 and 6 months post-CXL explained by initial partial endothelial damage followed sliding of undamaged endothelium in the area of the damaged endothelium. The utilization of the pulsed accelerated CXL protocol in our study could elucidate the less harmful effect of CXL on the corneal endothelial cell parameters compared with the continuous accelerated CXL protocol used in Cingü et al. and Badawi studies. An experimental study conducted by Zhu et al. [21] found that the pulsed-light accelerated CXL protocol was less injurious with faster recovery of ECD than the continuous-light accelerated CXL protocol in rabbit corneas. The higher mean preoperative endothelial cell count in our study is another possible factor interpreting the safer outcome. Bhandari et al. [22] documented that the changes in endothelial cell count and coefficient of variance were statistically significant and persisted until the end of the 12-month follow-up period following cl-ACXL with intensive UVA irradiance of 30 mW/cm^2^ for 3 min. A possible interpretation of their findings is the use of more intense UVA irradiance (30 mW/cm^2^) in a continuous mode. Another consideration is the possible statistical bias resulting from two patients in their study who showed high percentage of endothelial cell loss of 4.6% and 4.8%.

We explored a correlation between preoperative pachymetry and postoperative endothelial cell loss after pl-ACXL. A negative correlation was observed but it was of no statistical significance. Corneal thickness might not be the only factor responsible for corneal endothelial damage after cross-linking procedure. Improper calibration, inaccurate focusing and the infrequent instillation of riboflavin may be other significant factors worthy to be considered as suggested by Gokhale [23] who reported a 37-year-old male with a preoperative pachymetry above 400 μm presented with corneal edema and severe stromal haze one month after conventional epithelium-off CXL. Specular microscopy revealed that ECD was 1776 cells /mm^2^ which is below the ECD in the other untreated eye by 40%.

The mean demarcation line depth, as measured by AS-OCT, 1-month following pl-ACXL in our study was 214 ± 17.43 μm denoting a good penetrability into the corneal stroma similar to what was suggested by previous studies utilizing pulsed-light accelerated CXL protocol for management of keratoconus [24–26].

Recent systematic literature reviews [1, 27, 28] found that the clinical outcomes and safety of conventional and accelerated CXL protocols were comparable. In addition, pulsed mode may increase the long-term efficacy and safety of the accelerated protocols.

Our study had some notable limitations such the small sample size, the short follow-up period and the lack of comparison with other CXL protocols. Furthermore, it should be taken into consideration that the quality of images captured by the specular microscope and thus their interpretation after pl-ACXL might by affected by post-CXL haze (assessed by corneal densitometry), stromal keratocytes apoptosis and postoperative stromal edema especially in the early postoperative period (1–3 months) as reported in a study evaluating microstructural analysis of corneal layers following conventional and accelerated CXL by in vivo confocal microscopy (IVCM) [29].

## Conclusions

Corneal endothelial changes following high-fluence pl-ACXL for patients with mild and moderate keratoconus were minimal with stability of endothelial cell count and non-significant morphological changes. Further studies are needed to confirm the safety of high-fluence ACXL on corneal endothelium focusing on soaking time, irradiance level, and timing of UVA on/off period.

## Data Availability

Data are available from the corresponding author on reasonable request.

## List of abbreviations

KC: Keratoconus
CXL: Corneal collagen cross-linking
Pl- ACXL: Pulsed light accelerated corneal collagen cross-linking
Cl- ACXL: Continuous light accelerated corneal collagen cross-linking
UVA: Ultraviolet A-rays
ECD: Endothelial cell density
CV: Coefficient of variation
Kmax: Maximum keratometry
UDVA: Uncorrected distance visual acuity,
CDVA: Corrected distance visual acuity,
logMAR: Logarithm of the minimum angle of resolution
MRSE: Manifest refraction spherical equivalent
BSS: Balanced salt solution
NSAID: Non-steroidal anti-inflammatory drugs
AS-OCT: Anterior segment ocular coherence tomography
IVCM: In vivo confocal microscopy
